# Determining Soil Microbial Communities and Their Influence on *Ganoderma* Disease Incidences in Oil Palm (*Elaeis guineensis*) via High-Throughput Sequencing

**DOI:** 10.3390/biology9120424

**Published:** 2020-11-27

**Authors:** Yit Kheng Goh, Muhammad Zarul Hanifah Md Zoqratt, You Keng Goh, Qasim Ayub, Adeline Su Yien Ting

**Affiliations:** 1 School of Science, Monash University Malaysia, Bandar Sunway 47500, Malaysia; yit.goh@monash.edu (Y.K.G.); qasim.ayub@monash.edu (Q.A.); 2 Advanced Agriecological Research Sdn. Bhd., Petaling Jaya 47810, Malaysia; gohyk@aarsb.com.my; 3 Monash University Malaysia Genomics Facility, Bandar Sunway 47500, Malaysia; muhammad.zarulhanifah@monash.edu

**Keywords:** basal stem rot, disease incidence, microbiome, suppressive soil

## Abstract

**Simple Summary:**

Biological and physicochemical soil factors involved in the incidence of the basal stem rot (BSR) disease in an oil palm (*Elaeis guineensis*) plantation in Malaysia were characterized. Blenheim soil with a low BSR disease incidence and Bernam soil with high BSR disease incidence were analyzed and observed to have differences in composition and diversity of soil prokaryotic and eukaryotic communities. Blenheim soil with a high pH and calcium was shown to have higher prokaryotic and eukaryotic diversity compared to Bernam soil. High abundances of rare metabolically diverse and versatile bacterial taxa, bacterial taxa that increased with the introduction of biocontrol agents, potential disease-suppressive bacteria, and bacterivorous flagellates were observed in Blenheim soil. In contrast, Bernam soil was predominantly characterized by potential disease-inducible bacterial taxa. A combination of both abiotic and biotic elements might be essential in driving disease-suppressive soil microbiome toward *Ganoderma* BSR in Blenheim soil.

**Abstract:**

Basal stem rot (BSR), caused by *Ganoderma boninense*, is the most devastating oil palm disease in South East Asia, costing US$500 million annually. Various soil physicochemical parameters have been associated with an increase in BSR incidences. However, very little attention has been directed to understanding the relationship between soil microbiome and BSR incidence in oil palm fields. The prokaryotic and eukaryotic microbial diversities of two coastal soils, Blenheim soil (Typic Quartzipsamment—calcareous shell deposits, light texture) with low disease incidence (1.9%) and Bernam soil (Typic Endoaquept—non-acid sulfate) with high disease incidence (33.1%), were determined using the 16S (V3–V4 region) and 18S (V9 region) rRNA amplicon sequencing. Soil physicochemical properties (pH, electrical conductivity, soil organic matter, nitrogen, phosphorus, cation exchange capacity, exchangeable cations, micronutrients, and soil physical parameters) were also analyzed for the two coastal soils. Results revealed that Blenheim soil comprises higher prokaryotic and eukaryotic diversities, accompanied by higher pH and calcium content. Blenheim soil was observed to have a higher relative abundance of bacterial taxa associated with disease suppression such as Calditrichaeota, Zixibacteria, GAL15, Omnitrophicaeota, Rokubacteria, *AKYG587* (Planctomycetes), *JdFR-76* (Calditrichaeota), and *Rubrobacter* (Actinobacteria). In contrast, Bernam soil had a higher proportion of other bacterial taxa, Chloroflexi and *Acidothermus* (Actinobacteria). *Cercomonas* (Cercozoa) and *Calcarisporiella* (Ascomycota) were eukaryotes that are abundant in Blenheim soil, while *Uronema* (Ciliophora) and mammals were present in higher abundance in Bernam soil. Some of the bacterial taxa have been reported previously in disease-suppressive and -conducive soils as potential disease-suppressive or disease-inducible bacteria. Furthermore, *Cercomonas* was reported previously as potential bacterivorous flagellates involved in the selection of highly toxic biocontrol bacteria, which might contribute to disease suppression indirectly. The results from this study may provide valuable information related to soil microbial community structures and their association with soil characteristics and soil susceptibility to *Ganoderma*.

## 1. Introduction

Oil palm (*Elaeis guineensis* Jacq.) is a highly valuable commercial oil crop in South East Asia (SEA) [1,2]. Malaysia has the second largest acreage in the world dedicated to oil palm area (~5.8 million hectares), producing more than 19.5 million tons of palm oil [3] and contributing ~USD$16 billion to Malaysian export revenue [4]. This perennial crop is unfortunately susceptible to several pests and diseases. Basal stem rot (BSR) disease, caused by *Ganoderma boninense* Pat. (synonym: *Ganoderma orbiforme* (fr.) Ryvarden), is the most prevalent fungal disease of oil palm in SEA [5,6,7]. BSR disease causes rotting of the bole of the oil palm trunk, leading to the reduction of fruit production and incurring losses amounting to US$500 million annually in SEA [2,8,9]. There are several soil abiotic factors associated with high BSR disease incidences. For example, field observations revealed that low soil pH, high salinity, and high electrical conductivity, as well as heavy textured soils with poor drainage or with high water retention capacity, were associated with higher BSR incidence [5,10,11,12].

To date, there is no effective control measure known to prevent BSR disease [12]. Surgery, constructing isolation trenches, and soil mounding were studied and found to be tedious, expensive, and ineffective [8,13]. Sanitation approaches during existing plantings and at replanting have been practiced to minimize the size of diseased stumps, and have been known to reduce BSR disease incidences when done properly [13]. Fungicide drenching and trunk injection were generally not effective in controlling BSR disease [13]. These chemical approaches also cause environmental and safety issues. Furthermore, the development of effective and less laborious control of *Ganoderma* disease has been hindered by the lack of reliable early detection tools [8,12]. Therefore, this has encouraged the emphasis on environmental-friendly alternatives to manage BSR disease, which include biological control, development of BSR-tolerant or -resistant oil palm cultivars, and application or study of BSR-suppressive soil. Various potential fungal and bacterial biocontrol agents, namely *Trichoderma harzianum*, *T. viride*, *Pseudomonas fluorescens*, *P. aeruginosa*, *Burkholderia cepacia*, and *Bacillus* species, have been screened and studied for the control of *Ganoderma* disease in nursery, glasshouse, and field experiments [14,15,16]. In addition, biological control agents with plant growth promoting and other important antifungal traits, in particular *T. asperellum* and *P. aeruginosa* with chitinase, cellulase, glucanase, and indole acetic acid-producing capabilities, were studied for the control of BSR disease, improving the growth of the oil palm and facilitating better nutrient uptake by the plant [17]. Screening of various oil palm progenies and varieties from various origins, as well as development of disease-tolerant, or -resistant, cultivars were explored as well. For instance, Dura × Pisifera cross from Congo x Cameroon origin and Deli × Yangambi cultivars were reported with partial resistance to BSR disease [18,19,20].

Soil microbiota is critical in establishing healthy soil, improving soil fertility, and ensuring sustainable plant productivity. Microbial communities achieve these through mechanisms such as plant-growth promoting and stress-ameliorating capabilities, as well as suppressing soil-borne pathogens [21,22,23,24,25]. In a few instances, high microbial richness, diversity, and functional diversity of soil microbiota were reported to contribute to disease suppression and survival of the plants [24,26]. Soil suppressiveness, a plant defense mechanism established by native microorganisms in soil, provides the first defensive barrier against soil-borne phytopathogens [23,27]. Advancement in high-throughput second-generation sequencing has offered a new avenue in studying and characterizing soil microbiota that is related to disease-suppressive soils [25] and has since been applied to study soils suppressiveness towards *Pythium irregulare* [28], Fusarium wilt [29], and *Rhizoctonia solani* [27]. Ros et al. [28] reported that soil suppressiveness towards *P. irregulare* was mainly correlated to abiotic elements (pH, electrical conductivity, and total organic carbon), as well as biotic properties (bacterial and fungal taxa). Siegel–Hertz et al. [19] discovered that fungal and bacterial genera with known inhibitory activities toward phytopathogens were more abundant in suppressive soil. Mendes et al. [20] showed that γ- and β-Proteobacteria, Firmicutes, and Actinobacteria were more abundant in soils suppressive towards *R. solani*. Although the microbiomes of a wide range of disease-suppressive soils for crops has also been reported, the microbiome analysis of soils related to BSR disease of oil palm is not well characterized. Existing studies revealed that most of the 16S- and 18S-targeted amplicon sequencing studies related to Malaysian and Indonesian oil palm ecosystems compared soil microbiomes to understand the effects of land use. This included soils from rainforest, logged forest, rubber field, and oil palm cultivation [30,31,32,33]. None of the studies examined the microbiome of soils in relation to BSR incidence. Therefore, this study, embarking on the microbiome comparison between BSR-suppressive and -conducive soils, is expected to contribute immensely to the understanding of the microbial diversity and influence of microbiota on disease incidence of BSR.

Malaysian coastal soils have a long-established history of BSR [34]; unfortunately, information related to soil prokaryotic and eukaryotic microbial communities and diversity for oil palms planted on coastal soils is still limited. Blenheim and Bernam soils are coastal soils from an oil palm plantation in Perak, Malaysia. Blenheim soil (Typic Quartzipsamment—old beach ridge with shells) is characterized by coarse sand to loamy coarse sand texture and high pH, whereas Bernam soil (Typic Endoaquept—non-acid sulfate) is a silty clay to silty clay loam textured soil with low pH [35,36]. Both soils are planted with palms of similar planting material (Dura x Pisifera) and of the same age, and with similar management practices (i.e., inter-cropping, fertilizing routines) for the past two decades. Based on the initial census conducted in early 2018, BSR disease incidences in Blenheim soil were lower (less than 5%) compared to Bernam soil (more than 15%). In our study, we aimed to describe prokaryotic and eukaryotic communities of the two coastal soils from the oil palm plantation by using Illumina 16S rRNA and 18S rRNA amplicon sequencing, respectively. In addition, soil physicochemical parameters and the BSR incidence for palms planted on Blenheim and Bernam soils were recorded. To observe spatial variation between soil samples within close proximity, soil samples from four separate microsites, in particular top- and subsoils from palm circle and inter-palms regions were also included in the current study. Our findings provide new insights into the potential biotic and abiotic properties associated with soil suppressiveness of *Ganoderma* BSR disease in oil palm.

## 2. Materials and Methods

### 2.1. Site and Soil Collection

**Figure 1:**
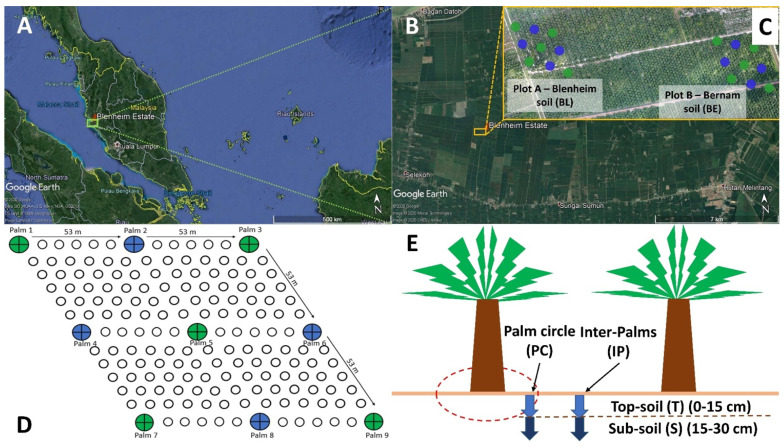
Location of Blenheim oil palm-coconut estate in northern peninsular Malaysia. (**A**) Geographic location. (**B**) Aerial view of experimental plots. (**C**) Plots A and B refer to selected blocks with Blenheim and Bernam soils, respectively. Soil sampling points are shown along the 53 × 53 m grid-line section. (**D**) Sample collection sites in each plot. Green- and blue-colored dots represent palm points that were selected for soil physicochemical analyses (*n* = 9). Only green-colored palm points were used for microbiome analyses (*n* = 5). (**E**) Four different microsites (topsoil (0–15 cm from soil surface) and subsoil (>15–30 cm from soil surface) from palm circles (PC) and inter-palms (IP)) were selected for soil sampling. Abbreviations: T—Topsoil and S—Subsoil.

An oil palm estate in Perak, Malaysia (Figure 1A), which was planted in 1998 with Tenera (Dura × Pisifera) oil palms in an equilateral triangle planting system (planting distance of 8.80 × 8.80 × 8.80 m), was selected for this study. The estate experiences a tropical climate with annual precipitation ranging from 1700 to 2300 mm (Supplementary Materials Table S1). Two palm plots, approximately 1 km apart, were located on Blenheim (coordinates: 3°55′44.6″ N, 100°48′36.2″ E) (Plot A) and Bernam (coordinates: 3°55′33.4″ N, 100°48′51.7″ E) (Plot B) coastal soils, respectively (Figure 1B). These soils are classified as Typic Quartzipsamment and Typic Endoaquept, respectively, according to the classification systems by USDA [35,36]. These soil types were selected for the study due to their different susceptibility towards BSR disease despite their relatively close proximity, and were subjected to typical cultural management practices and fertilizer regimes (Supplementary Materials Table S2).

Soil sampling was conducted in May and December 2018 for soil physicochemical and microbiome analyses (Supplementary Materials Figure S1A,B). Sampling points were based on grid-line sections (intervals of 53 × 53 m), and a total of 9 sampling points of the respective soil types (within an area of 1.06 ha) were selected (Figure 1B,C). Soil samples from all the 9 palm points (9 replicates) (Figure 1B,C—green- and blue-colored solid circles) were collected from palm circle (PC) and inter-palm (IP) regions at depths of 0–15 (topsoil) and 15–30 (subsoil) cm (Figure 1D) using Dutch Auger, and sent to the Advanced Agriecological Research (AAR) Chemistry Laboratory for physicochemical analyses.

For soil microbiome analysis, only five out of nine palms were selected (Figure 1B,C—green-colored solid circles). At the respective sampling point, soil trenches were dug next to the auger point to collect soil samples from four separate microsites (Figure 1D). In May 2018, soil from ten selected palm points (five for Blenheim and five for Bernam) were analyzed. Four separate microsites were sampled across each of these palm points and pooled together for analysis (Figure S1A) (2 soil types × 1 composite of microsites × 5 sampling points = 10 soil samples). In December 2018, a modified sampling strategy was adopted in which forty soil samples (twenty from each soil type) were collected from four separate microsites (Supplementary Materials Figure S1B) of the respective sampling points (Figure 1B,C—green-colored solid circles) (2 soil types × 4 microsites × 5 sampling points = 40 soil samples). Microsites were sequenced separately only for December 2018 samples (Supplementary Materials Figure S1B) to determine whether there were any differences in the prokaryotic and eukaryotic communities between the different microsites for each soil type. Soils were transported back to the laboratory on ice. Soil samples were broken into small pieces and homogenized manually using a sterilized spatula [37]. Plant debris, intact shell debris, and roots were removed using sterilized forceps before sieving through a 2-mm sieve. The soils were then kept at −80 °C until DNA extraction.

### 2.2. Field BSR Disease Census

BSR disease incidence and severity were recorded every six months. Key indicators of BSR incidence were based on visual symptoms that were subsequently used to determine the disease index. Disease classes adopted for calculating disease severity index were described previously by Chen et al. [38], with slight modifications as follows: (a) Healthy (class 0); (b) *Ganoderma* fruiting body or basidiocarp (class 1); (c) rotten bole (class 2); (d) combination of *Ganoderma* fruiting body or basidiocarp and rotten bole (class 3); and (e) vacant point with sanitation (class 4). Disease incidence (DI) was determined using the formula outlined below [39], where *n* is the number of palms identified as diseased; and *N* is the total number of censused palms. DI refers to the percent of palms within the plots that demonstrated visual infection symptoms (disease classes 1 to 4).

(1) $$\mathrm{DI}=\frac{n}{N}\times 100\%$$

Disease severity index (DSI) was calculated based on the formula illustrated below [40], where *N* is the total number of censused palms, and *ni* refers to the number of palms categorized into category *i* (based on disease class 0 to 4). DSI indicates the level of disease severity for the infected palms within the plots.

(2) $$\mathrm{DSI}=\sum \frac{\left(i\times ni\right)}{\left(4\times N\right)}\times 100$$

### 2.3. Soil Physicochemical Analyses

Soil pH was determined using 1 M potassium chloride (KCl) (2:5—soil to solution ratio) solution with IQ Scientific 150 pH meter (Spectrum Technologies, Plainfield, IL, USA) [41]. Electrical conductivity (EC) was assessed with an EC meter (S30, Mettler Toledo, Columbus, OH, USA) [42]. The percentage of soil organic matter was quantified using the loss-on-ignition method with muffle furnace (L9/11C6, Nabertherm, Lilienthal, Germany) [43]. Total nitrogen (N) (Gerhardt distiller, VAP45S, Cologne, Germany), available phosphorus (AP), and total P (TP) content of the soil were determined using micro-Kjeldahl, Bray-2, and 6 M HCl extraction methods, respectively [44,45,46]. AP and TP were quantified using a UV-Visible Spectrophotometer (Evolution 201, Thermo Scientific, Waltham, MA, USA). Cation exchange capacity (CEC) was quantified using 1 M ammonium acetate (pH 7.0), and the extracted soil leachates were quantified for exchangeable potassium (K) and sodium (Na) (with flame photometer) (M410, Sherwood Scientific, Cambridge, UK), and CEC and magnesium (Mg) (with atomic absorption spectrometer or AAS) (AA100, Pelkin Elmer, Shelton, CT, USA) [43,45,46,47]. Soil micronutrients, namely iron (Fe), manganese (Mn), zinc (Zn), copper (Cu), and calcium (Ca) were analyzed with AAS, and silica (Si) was determined with inductively coupled plasma-optical emission spectrometry (Optima 7300DV, Pelkin Elmer, Shelton, CT, USA) in accordance with the procedures outlined previously [43,48]. Soil physical properties were determined using hydrometer (Zeal, London, UK) [43].

### 2.4. Soil DNA Extraction, Library Preparation, Purification, and MiSeq Sequencing

A soil sample (0.3 g) was used for soil DNA extraction using the DNeasy PowerSoil Kit (Qiagen, Valencia, CA, USA) as per the manufacturer’s instruction. For each sample, three extractions were performed, and the extracted DNAs were pooled into one composite [49]. The hypervariable V3–V4 region of 16S rRNA gene was amplified using Bakt 341F (5′-CCT ACG GGN GGC WGC AG-3′) and Bakt 805R (5′-GAC TAC HVG GGT ATC TAA TCC-3′) primers comprising partial Illumina Nextera adapter at the 5′ end [50,51]. 1391F (5′-GTA CAC ACC GCC CGT C-3′) and EukBr (5′-TGA TCC TTC TGC AGG TTC ACC TAC-3′) primers [52,53] containing partial Illumina adapter at the 5′ end were used to target the hypervariable V9 region of the 18S rRNA gene [54]. Fragments of V3–V4 (16S rRNA) and V9 (18S rRNA) regions were amplified with the HotStar HiFidelity DNA Polymerase Kit (Qiagen, Valencia, CA, USA) [55] using Mastercycler^®^ nexus GSX1 (Eppendorf, Hamburg, Germany), set to the PCR (polymerase chain reaction) conditions described previously [56]. Amplified amplicons were purified with the Agencourt AMPure XP bead (Beckman Coulter, Brea, CA, USA) before indexing with Illumina Nextera XT Index i5 and i7 using the KAPA HiFi HotStart ReadyMix Kit (KAPA BioSystems, Woburn, MA, USA). Indexed 16S and 18S amplicons were purified and quantified using the Qubit^®^ dsDNA HS assay with Qubit^®^ 2.0 Fluorometer (Invitrogen, Carlsbad, CA, USA). The 16S and 18S libraries were further quantified, quality checked, normalized, and processed [57] prior to sequencing with the Illumina MiSeq (Illumina, San Diego, CA, USA) at the Monash University Malaysia Genomics Facility with a 2 × 250 bp run configuration.

### 2.5. Sequence Processing and Analyses

Trimmomatic was used to trim poor quality bases within 20 base pairs from 3′ end [58]. Primers targeting V3–V4 and V9 regions of 16S and 18S rRNA, respectively, were excised from the trimmed pair-end sequences using Cutadapt [59]. Trimmed forward and reverse sequences of 16S and 18S rRNA were merged using Usearch10 [60]. Microbiome bioinformatics for the merged 16S and 18S rRNA sequences were then analyzed using QIIME 2 2019.4 [61]. Merged reads were denoised using DADA2 (with q2-dada2) [62]. All the observed features generated were aligned using mafft (with q2-alignment) [63], and fasttree2 (with q2-phylogeny) [64] was employed to construct a phylogenetic tree. Taxonomic assignment was performed with a q2-feature-classifier [65], which is a sklearn naïve Bayes taxonomy classifier trained [66] against the SILVA database (version 132—99% OTUs reference sequences). Observed features with no assignment were then re-classified against the SILVA SSU Ref database (version 132) using SINA 1.3.1 [67,68]. ASVs assigned to or identified as chloroplasts or mitochondria were removed from the representative sequences and feature table prior to subsequent analyses. Alpha-diversity (namely observed features and faith phylogenetic diversity (Faith PD) [69], as well as beta-diversity, in particular, weighted UniFrac [70,71]) metrics were determined using q2-diversity after normalization of all the samples to an equal sequencing depth (13,600 and 11,000 reads/sample for 16S and 18S rRNA, respectively). Observed features, Faith PD, and composition of the top 10 most abundant phyla and genera of the soil microbiomes were illustrated using the R ggplot2 package [72]. Raw sequences of 16S and 18S rRNA were submitted to the Sequence Read Archive (SRA) by the National Centre for Biotechnology Information (NCBI) with the BioProject accession number: PRJNA649668.

### 2.6. Statistical and Data Analyses

All soil physicochemical parameters of two coastal soils were analyzed with principal component analysis (PCA) in RStudio [73] to determine the variables that distinguished Blenheim from the Bernam soil types. A two-by-two chi-square test (at *p*-value ≤ 0.0025 after Bonferroni correction) was adopted to determine the differences in the number of infected and healthy palms planted on Blenheim and Bernam soil types.

Differences in alpha diversities (observed features and Faith PD) between soil types and across soil microsites for the respective soil types of 16S and 18S rRNA datasets were assessed using the Kruskal–Wallis test in QIIME 2 with a significance level at Benjamini–Hochberg *q*-value < 0.05 [74]. Principal coordinate analyses (PCoA) based on the weighted-UniFrac distance matrix were estimated with the ordination approach [70,75] and the PCoA plots were visualized in the R ggplot2 package. Non-parametric analysis of similarities (ANOSIM) incorporating the default number of permutations (999) (at Benjamini–Hochberg *q*-value of <0.05) was used to assess differences in soil microbial communities (beta diversity: weighted-UniFrac) between soil types and across soil microsites for the respective soil types of both 16S and 18S rRNA datasets in QIIME 2. Differential abundance of taxa between two soil types for 16S and 18S rRNA datasets was studied using analysis of composition of microbiomes (ANCOM) [76].

## 3. Results

### 3.1. Ganoderma Incidences in Blenheim and Bernam Soils

BSR disease incidence (DI) and disease severity index (DSI) in palms planted on Bernam soil was higher than Blenheim soil (Table 1). Both the DI and DSI recorded in the Bernam plot had increased significantly over the period of 18 months from May 2018 to December 2019. Palms assessed with the disease classes of 3 and 4 also increased in the Bernam plot. In addition, Bernam soil was also assessed with a significantly higher number of infected palms over healthy palms than Blenheim soil at all four census time points (two-by-two contingency table chi-square test *p* < 0.0001) (Table 2).

### 3.2. Physicochemical Analyses of Blenheim and Bernam Soils

**Figure 2:**
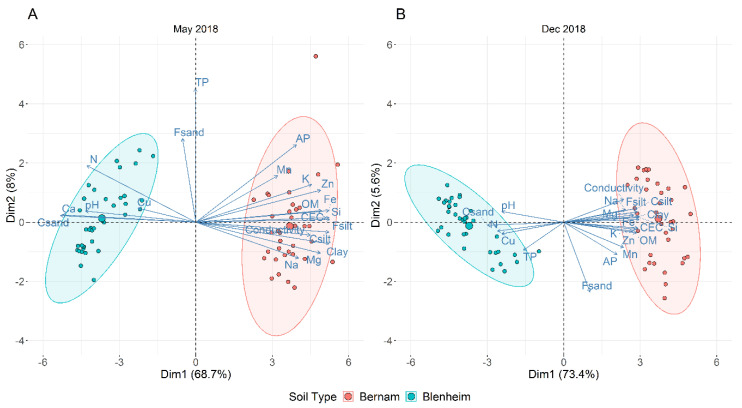
Principal component analysis (PCA) plot based on all the tested soil physicochemical parameters for Blenheim (BL) and Bernam (BE) soil samples collected in May (**A**) and December (**B**) 2018. Abbreviations of different physicochemical parameters: pH (pH measured in 1 M potassium chloride); OM (organic matters by loss-on-ignition), N (nitrogen), Fe (iron), Si (silica), TP (total phosphorus), AP (available phosphorus), Mn (manganese), Zn (zinc), Cu (copper), K (exchangeable potassium), Ca (calcium), Mg (exchangeable magnesium), Na (exchangeable sodium), CEC (cation exchange capacity), EC (electrical conductivity), Coarse sand (Csand), Fine sand (Fsand), Coarse silt (Csilt), and Fine silt (Fsilt).

Blenheim and Bernam soil samples of May and December 2018 have distinct physicochemical properties (Table 3 and Supplementary Materials Table S3). Principal component analysis (PCA) plots illustrated six soil physicochemical parameters, namely pH, N, Ca, Cu, TP, and coarse sand (CSand) (along the negative of x-axis), which distinguished Blenheim soil from Bernam soil, and the first component explained 67.4 to 74.5% of the variance, whereas component 2 explained 5.4 to 8.4% of the variance (Figure 2A,B).

### 3.3. Prokaryotic and Eukaryotic Richness and Diversity in Blenheim and Bernam Soils

**Figure 3:**
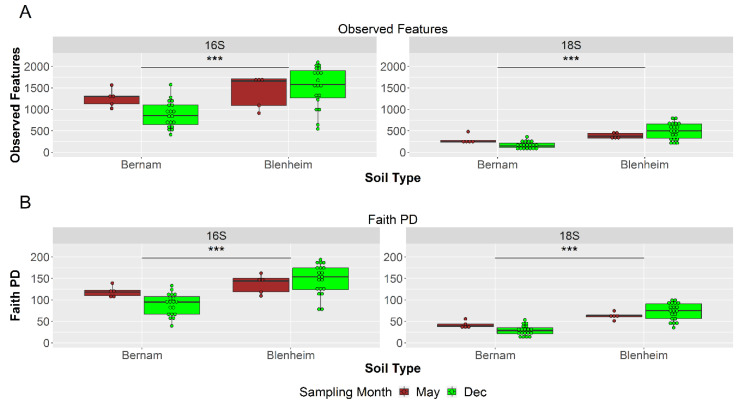
Alpha-diversity based on observed features (**A**) and Faith phylogenetic diversity (Faith’s PD) (**B**) of soil prokaryotic (16S) and eukaryotic (18S) communities for Blenheim and Bernam soils collected in May (brown-colored boxplot) and December (green-colored boxplot) 2018. Asterisk of *** denote significance between soil types at *p* < 0.001 after Kruskal–Wallis test.

Bernam soil showed significantly lower richness and diversity (observed features and Faith PD) of prokaryotic and eukaryotic communities compared to Blenheim soil (*p*-value < 0.001; Kruskal–Wallis test) (Figure 3 and Supplementary Materials Figure S2). Comparison of richness and diversity between microsites of prokaryotic communities of Bernam soil or Blenheim soil (except for the Faith PD between top- and subsoils of inter-palms within Blenheim soil), as well as eukaryotic community of Bernam soil were not significantly different (*q*-value ≥ 0.05; Kruskal–Wallis test) (Supplementary Materials Figure S3A,B). However, richness and diversity of eukaryotic communities were significantly higher in the topsoil inter-palms (IPT) microsite of Blenheim soil compared to its subsoil inter-palms (IPS) (*q*-value = 0.02; Kruskal–Wallis test) (Supplementary Materials Figure S3A,B). In summary, Blenheim soil had a higher prokaryotic and eukaryotic richness and diversity compared to Bernam soil.

### 3.4. Prokaryotic Microbial Community at Phylum and Genus Levels

The majority of the reads from Blenheim and Bernam soils were taxonomically assigned to members from the top 10 dominant phyla, in particular Proteobacteria, Firmicutes, Acidobacteria, Chloroflexi, Bacteroidetes, Verrucomicrobia, Actinobacteria, Rokubacteria, Planctomycetes, and Nitrospirae (Supplementary Materials Figure S4A). Furthermore, the results also revealed that the ten most abundant genera from Blenheim and Bernam soils were MSBL7 (Proteobacteria, Desulfobulbaceae), *Neisseria* (Proteobacteria, Neisseriaceae), *Paraliobacillus* (Firmicutes, Bacillaceae), *Acidovorax* (Proteobacteria, Burkholderiaceae), *Dysgonomonas* (Bacteroidetes, Dysgonomonadaceae), *Paenibacillus* (Firmicutes, Paenibacillaceae), *Mariprofundus* (Proteobacteria, Mariprofundaceae), *Nitrospira* (Nitrospirae, Nitrospiraceae), *Fulvivirga* (Bacteroides, Flammeovirgaceae), and *Candidatus* Udaeobacter (Verrucomicrobia, Chthoniobacteraceae) (Supplementary Materials Figure S4B).

**Figure 4:**
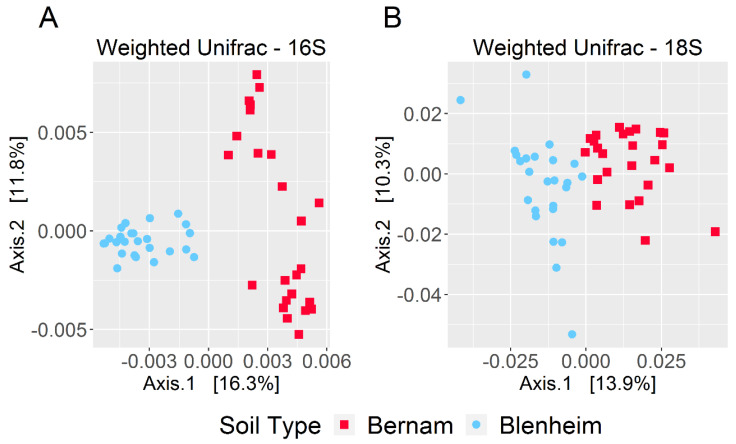
Beta-diversity of prokaryotic and eukaryotic communities. Principal coordinates analysis (PCoA) plots based on weighted-UniFrac matrices for prokaryotic (**A**) and eukaryotic (**B**) microbiomes.

**Figure 5:**
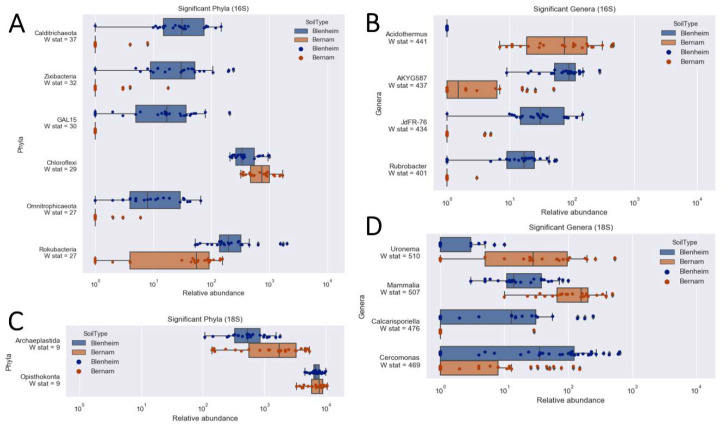
Relative abundances of significant prokaryotic (**A**,**B**) and eukaryotic (**C**,**D**) taxa for Blenheim and Bernam soils at phylum/supergroup (**A**,**C**) and genus (**B**,**D**) levels.

Based on weighted-UniFrac diversity, prokaryotic communities between Bernam and Blenheim soils were significantly different (ANOSIM test, 999 permutations, *p*-value = 0.001) (Figure 4A). At the phylum level, Calditrichaeota, Zixibacteria, GAL15, Omnitrophicaeota, and Rokubacteria were significantly more abundant in Blenheim soil compared to Bernam soil (with W stat = 37, 32, 30, 27, and 27, respectively) (Figure 5A). On the contrary, Chloroflexi phylum was significantly higher in Bernam soil compared to Blenheim soil (W stat = 29) (Figure 5A). At the genus level, relative abundances of *AKYG587* (Planctomycetes), *JdFR-76* (Deferribacteres), and *Rubrobacter* (Actinobacteria) genera were significantly higher in Blenheim soil compared to Bernam soil (with W-stat = 437, 434, and 401, respectively), whereas *Acidothermus* genus was significantly higher in Bernam soil (W-state = 441) (Figure 5B). The results showed that the abundance of a few unique phyla and genera were significantly higher in Blenheim soil compared to Bernam soil.

### 3.5. Eukaryotic Microbial Community Structure at Supergroup and Genus or Class Levels

The majority of the reads from Blenheim and Bernam soils were taxonomically assigned to members from the top nine supergroups of Eukaryota, in particularly Opisthokonta, SAR, Archaeplastida, Amoebozoa, Excavata, Cryptophyceae, Centrohelida, *Incertae sedis* Eukaryota, and Haptophyta (Supplementary Materials Figure S4C). In addition, the 10 most abundant eukaryotic genera, classes, or orders (Phylum) detected in Blenheim and Bernam soils were *Cercomonas* (Cercozoa), *Acanthamoeba* (Amoebozoa), Silicofilosea (Cercozoa), Conthreep (SAR), *Platyamoeba* (Amoebozoa), Sphaeropleales (Chlorophyta), Coccidia (Apicomplexa), *Vannella* (Amoebozoa), *Gregarinasina* (Apicomplexa), and Euglenida (Euglenozoa) (Supplementary Materials Figure S4D).

Eukaryotic communities of Blenheim and Bernam soils were significantly different (ANOSIM test, 999 permutations, *p*-value = 0.001) (Figure 4B). At the supergroup level, Archaeplastida and Opisthokonta were significantly higher in Bernam soil compared to Blenheim soil (with W-stat = 9) (Figure 5C). At the genus level, *Cercomonas* (Phylum: Cercozoa; order: Cercomonadida) was more dominant in Blenheim and *Uronema* (Phylum: Ciliophora; order: Conthreep) was more abundant in Bernam soil (W-stat = 628 and 683, respectively) (Figure 5D). The *Calcarisporiella* (Phylum: Zygomycota; order: Calcarisporiellales) genus was more prevalent in Blenheim soil. Mammal communities were more abundant in Bernam soil (W-state = 507) (Figure 5D).

Interestingly, there were no observed features assigned to the genus *Ganoderma,* with low observed features of the order Polyporales (Phylum: Basidiomycota) detected in the rarefied datasets. In the non-rarefied raw sequences, there were also low observed features of *Ganoderma.*

## 4. Discussion

Blenheim soil was identified with higher prokaryotic and eukaryotic richness and diversity, and both the prokaryotic and eukaryotic communities were different between two soils. It is postulated that the higher microbial diversity in Blenheim soil may have been attributed to their high soil pH (around 7 to 8), high calcium content (calcareous nature), and light textural class. These abiotic factors typically indicate microbial preference for near-neutral conditions, illustrating an increase of bacterial and eukaryotic diversity and richness, as well as microbial biomass [77,78,79]. Soil pH has always been implicated as one of the important soil abiotic elements influencing soil microbiota diversity and richness [80]. Bacterial diversity and relative abundance were greater in alkaline soil (pH 8) compared to acidic soil (pH 4) [81]. In addition, diversity of bacterial communities was also higher in the soil derived from calcareous parent material compared to soil originated from siliceous parent material, and bacterial compositions of the soils from two distinct parent materials were also significantly different [63]. Increasing evidence has elucidated the importance of high microbial diversity in reducing soil-borne diseases or contributing to soil suppressiveness [65,66,67,68] and high microbial diversity has been hypothesized to augment the functional diversity in the soil [69]. In addition, soil microbial diversity was proposed to correlate positively with pathogen resistance in plants [79]. A recent study in Sabah, Malaysia, showed that higher bacterial diversity was observed in disease-free soils compared to soils with high BSR incidence [13]. While this suggested that higher bacterial diversity may be associated with BSR disease suppression, more studies are required to ascertain their role and impact on disease suppression. Acidic or low soil pH with high concentration of aluminum ions are the major inherent characteristics of the marine alluvial Bernam soil type [82]. Low soil pH or acidic soil was observed to reduce rhizobia’s activity due to aluminum toxicity, affect the efficacy of siderophore-producing biocontrol bacteria, and decrease the growth, activity, and disease suppression by *P. fluorescens* and *Bacillus cereus* [83,84,85]. Low soil pH has also been reported to favor the growth and antagonistic activity of Trichoderma species compared to alkaline pH [86,87]. Unfortunately, acidic soil and low pH conditions also allow *G. boninense* to thrive and contribute to higher *Ganoderma* disease severity and incidences as compared to a more neutral pH 6 to 7 [88,89].

A few rare and unique phyla, such as Calditrichaeota, Zixibacteria, GAL15, Omnitrophicaeota, and Rokubacteria, distinguished Blenheim soil from Bernam soil. Rare Calditrichaeota, Zixibacteria, GAL15, Omnitrophicaeota, and Rokubacteria phyla were described as candidate phyla radiation (CPR) through culture-independent approaches, and are relatively less explored candidate divisions [90,91,92,93,94]. This study also presents the first observation of rare Calditrichaeota, Zixibacteria, Omnitrophicaeota, and Rokubacteria in shell deposit calcareous soil (Blenheim soil). Among five of the CPR observed in Blenheim soil, Zixibacteria and Rokubacteria phyla, the most explored CPR genomically were shown to be highly versatile in metabolism (e.g., iron reducing-oxidation and other metabolic pathways) and equipped with genes that encode for antimicrobial secondary metabolites or peptides (e.g., polyketide and non-ribosomal peptide synthetases), respectively [91,95]. Microbiomes with a high abundance of functional genes encoding for antimicrobial and antibiotic compound production are known to confer protection to plants, as observed in disease suppression towards *Ralstonia solanacearum* by *Alphaproteobacteria, Firmicutes,* and *Cyanobacteria* phyla, as well as *Pseudomonas* and *Bacillus* genera [24]. Furthermore, Calditrichaeota phylum was studied through a culture-independent and genomic approach and described to be a potential detrital protein degrader with exogenous peptidases [90]. Detection of significantly higher abundance of GAL15 and Omnitrophicaeota phyla, and JdFR-76 genus (Phylum: Calditrichaeota) in Blenheim soil, could be due to various environmental factors reported previously, namely poorer soil nutrient status or less fertile soil, soil originated from calcareous parent material, and high soil pH (around pH 7) [96,97,98,99,100].

*Rubrobacter* (Phylum: Actinobacteria), AKYG587 (Phylum: Planctomycetes), and JdFR-76 (Phylum: Calditrichaeota) genera were more abundant in Blenheim soil. *Rubrobacter* was reported as one of the more prevalent genera in both *Fusarium* wilt suppressive soil and strong *Fusarium graminearum* fungistatic natural soil [29,101,102,103]. AKYG587 was observed with higher relative abundance when either *Pseudomonas* or *Bacillus* biocontrol agents (BCA) were being inoculated [104,105]. AKYG587 could be a potential helper, symbiont, or mutualist for improving the efficacy of BCA in the soil. Further research is required to verify these associations. The existence of significantly higher relative abundances of rare and highly metabolic versatile bacterial CPR in Blenheim soil might improve *Ganoderma* disease suppressiveness. On the contrary, Chloroflexi dominated Bernam soil. Chloroflexi was illustrated as a tobacco disease-promoting bacterial phylum [106] and was more prevalent in disease-conducive soils [107,108]. Furthermore, the relative abundance of Chloroflexi was reduced with the introduction of BCA [104,105]. In addition, Bernam soil is also dominated with *Acidothermus* (Phylum: Actinobacteria) too. *Acidothermus* was found to be more dominant in disease-conducive soil [107] and also significantly higher in arbuscular mycorrhizal fungi suppressive soil [109]. The existence of significantly higher relative abundances of Chloroflexi phylum and *Acidothermus* genus in Bernam soil might lead to *Ganoderma* disease conduciveness and also affect the proliferation of beneficial microbes.

*Cercomonas* (Phylum: Cercozoa; order: Cercomonadida) and *Calcarisporiella* (Phylum: Zygomycota; order: Calcarisporiellales) were more abundant in Blenheim soil, whereas *Uronema* (Phylum: Ciliophora; order: Conthreep) was more prevalent in Bernam soil. Members from *Cercomonas* genus were found to feed selectively on non-toxic or less toxic bacteria and allowed the proliferation of highly toxic bacterial BCAs [110,111]. *Cercomonas longicauda* and *Hartmannella vermiformis* were the only two tested protozoa not inhibited by *Pseudomonas fluorescens* CHA0 strain [112,113]. This allowed both *C. longicauda* and *H. vermiformis* to grow among the highly potent BCAs and selectively consume the less toxic bacteria. The selection of highly toxic bacterial BCA through selective feeding behavior of *Cercomonas* can be useful in shaping soil to be suppressive toward plant pathogens. In the current study, the relative abundance of *Acanthamoeba* genus was slightly higher in Blenheim soil compared to Bernam soil; however, it was not significant (W-stat = 324, null hypothesis was not rejected). In a previous study by Müller et al. [114], the abundancy of the bacterial isolates with biocontrol genes encoded for the production of antifungal 2,4-diacetylphloroglucinol and hydrogen cyanide metabolites was augmented upon introduction of *Acanthamoeba* protozoan into the soil. This phenomenon has been proposed to enhance the biocontrol ability and antagonism activity of these bacterial isolates through production of antimicrobial secondary metabolites. Environmental factors, namely soil pH, were found to affect the relative abundance of protozoa. Buyer et al. [115], through fatty acid markers, showed that Galestown soil (pH 5.8 and 0.6% humic material) had higher protozoa and bacteria compared to Hatborough (pH 4.5 and 3.2% humic material) soil. On the contrary, Hatborough soil had higher fungi and eukaryotes. In addition, high pH conditions were reported previously to increase the relative abundance of Cercozoa and Ciliophora protists as well [77]. The relative abundance of *Cercomonas* in Blenheim soil was higher compared to Bernam soil and this could be due to high soil pH in the former soil.

Members of *Calcarisporiella* were more abundant in Blenheim soil. *Calcarisporiella thermophila* and *Calcarisporiella*-related species were isolated and described from coal spoil tip soil, pineapple field soil, and subsoil with warm and hot climates [116]. The tropical climate in Malaysia might have contributed to the growth of *Calcarisporiella.* Unfortunately, very limited information related to *Calcarisporiella* species in high pH and Ca soil, isolated from the shell deposit environment, as well as disease-suppressive soil, is available. On the other hand, members of *Uronema*, more prevalent in Bernam soil, have been reported as common opportunistic pathogens for fishes [117] and also bacterial feeders [118]. However, there is limited information on members of the *Uronema* genus in relation to the selection of bacterial BCAs with high biocontrol ability or genes, and agricultural aspects. Furthermore, salinity and sodium chloride concentration have been implicated to affect the growth of flagellate *Cercomonas* and ciliate *Uronema* species. Growth of *Cercomonas* species was suppressed at the salinity of 5 to 10 parts per thousands (ppt) [119]. Optimum growth for marine *Uronema* ciliates was in the range of 17–43 ppt salinity [120]. Mammalian communities were more prevalent in Bernam soil and this could be due to higher density and coverage of understory vegetation (namely various ferns), more complex habitat, and the presence of natural shelters [121,122]. Proliferation of understory vegetations, especially ferns, were observed to be better in Bernam soil (low pH), and this could be due to clayey soil with better water-holding capacity and also higher water table. Furthermore, invertebrates in tropical soils were reported as highly abundant and generally more tolerant towards lower pH (between 3.8 to 4.0) [123], and could be a promising food source for mammals too. Maintenance of understory vegetation increases the abundance of aboveground invertebrate and other macrofauna communities, and also establishes a more complex food web within the oil palm ecosystem [124]. A more detailed study across multiple soil types with a wide range of *Ganoderma* BSR incidences and a more diverse soil physicochemical parameters will improve our understanding on the effects of specific biotic and abiotic factors on *Ganoderma* BSR disease in oil palm.

Observation of extremely low observed features matching to the genus *Ganoderma* might also be due to low inoculum level in the sampled sites. Growth of *Ganoderma* may be influenced by the low organic matter present, namely the palm circle (1 m from the palm base) and inter-palms (approximately 30 to 50 cm from the frond heap). Furthermore, *Ganoderma boninense* was shown to be a weak competitor and unable to grow well in soils with low or no organic matter [6]. Basidiomycetous *G. boninense* was also proposed to survive better only on wood debris/substrates and other organic matters compared to soil medium [6]. In a most recent study, *G. boninense* was found to be incapable of thriving and spreading out from the inoculation site into the soil medium [125]. We hypothesized that the absence of the features assigned to the *Ganoderma* genus could also be due to a low number of basidiospores present at the sampling points (approximately 1 m from the palm base).

## 5. Conclusions

The composition and diversity of soil prokaryotic and eukaryotic communities were distinctly different between Blenheim soil and Bernam soil. Blenheim soil was observed to comprise higher prokaryotic and eukaryotic diversities compared to Bernam soil. Blenheim soil, with high soil pH and Ca, was also found to have higher abundance of rare metabolically diverse and versatile Candidate phyla radiation (CPR) bacteria (e.g., Rokubacteria and Zixibacteria), potential disease-suppressive bacterial taxa (e.g., *Rubrobacter*), bacterial taxa that increased with the introduction of biocontrol agents (e.g., *AKYG587*), and bacterivorous flagellates for the selection of highly toxic biocontrol bacteria (e.g., *Cercomonas*). On the contrary, Bernam soil harbored potential disease-inducible bacteria, particularly Chloroflexi and *Acidothermus* (Actinobacteria), which are associated with disease-conducive soil. The existence of Calditrichaeota, GAL15, Omnitrophicaeota, *JdFR-76* (Calditrichaeota), and *Calcarisporiella* (Ascomycota) could potentially be due to the nature and properties of Blenheim soil. Detection of high-relative abundance of less explored and uncultured CPR in Blenheim soil warrants further research into their relationships with the soil physicochemical properties and low *Ganoderma* incidence. Soil pH, Ca, and other soil physicochemical parameters could shape or drive the differences in both prokaryotic and eukaryotic communities between two soils. High abundance of mammalian communities could be due to high density and coverage of understory vegetation (e.g., ferns), and presence of natural shelters in Bernam soil. In addition, low soil pH could potentially be promoting growth and activity of pathogenic *G. boninense* in the Bernam soil type. A combination of abiotic and biotic elements might be pivotal in driving disease-suppressive soil microbiome toward *Ganoderma* BSR in Blenheim soil.

## Figures and Tables

**Table 1:** *Ganoderma* disease scores in terms of disease incidence (%) and disease severity index on Blenheim and Bernam soils for 2018 and 2019 (May and December).

Disease Class ^§^	Scoring	Blenheim (Typic Quartzipsamment)				Bernam (Typic Endoaquept)			
		2018		2019		2018		2019	
		May	Dec	May	Dec	May	Dec	May	Dec
**Healthy (%)**	**0**	98.7	98.7	98.7	98.1	75.2	73.5	72.4	66.9
**FB (%)**	**1**	0	0	0	0	10.5	11.6	8.8	10.5
**Rot (%)**	**2**	0	0	0	0.6	1.1	1.1	1.7	1.1
**Rot + FB (%)**	**3**	0	0	0	0	5.5	5.5	7.7	11
**Vacant + Sanitation (%)**	**4**	1.3	1.3	1.3	1.3	7.7	8.3	9.4	10.5
**Total Palm Censused**		158	158	158	158	181	181	181	181
**Disease Incidence (%)**		*1.3*	*1.3*	*1.3*	*1.9*	*24.9*	*26.5*	*27.6*	*33.1*
**Disease Severity Index**		*1.27*	*1.27*	*1.27*	*1.58*	*15.06*	*15.88*	*18.23*	*21.96*

^§^ Abbreviations for the disease classes: FB—Fruiting body; Rot—Rotten stem bole; Rot + FB—combination of both rotten stem bole and fruiting body; and vacant + sanitation—Vacant sanitized palm point (after toppling of the diseased palms).

**Table 2:** A two-by-two contingency table for determining the number of infected palms (with the disease scores of 1 to 4) over healthy palms (with the disease score of 0) between Blenheim and Bernam soils on four census time points is shown below.

Year	Census Time Points	Soil Types	Status of the Palms			*p*-Value after Chi-Square Test
			No. of Infected Palms	No. of Healthy Palms	Total Assessed Palms	
2018	May	Blenheim	2	156	158	*<0.0001*
		Bernam	45	136	181	
	December	Blenheim	2	156	158	*<0.0001*
		Bernam	48	134	181	
2019	May	Blenheim	2	156	158	*<0.0001*
		Bernam	50	132	181	
	December	Blenheim	3	155	158	*<0.0001*
		Bernam	60	122	181	

**Table 3:** Soil chemical properties of Blenheim and Bernam soil series collected from four microsites in May and December 2018.

Chemical Parameters ^§^	Blenheim (Typic Quartzipsamment)								Bernam (Typic Endoaquepts)							
	May 2018				Dec 2018				May 2018				Dec 2018			
	PCT *	PCS *	IPT *	IPS *	PCT	PCS	IPT	IPS	PCT	PCS	IPT	IPS	PCT	PCS	IPT	IPS
pH	7.65 (0.05)	7.67 (0.06)	7.73 (0.07)	7.66 (0.06)	7.90 (0.08)	8.05 (0.10)	7.90 (0.08)	7.96 (0.08)	5.66 (0.42)	5.83 (0.41)	6.23 (0.23)	5.99 (0.35)	5.53 (0.48)	5.73 (0.37)	5.71 (0.43)	5.44 (0.50)
OM (%)	5.36 (0.57)	5.53 (0.38)	5.08 (0.66)	5.29 (0.52)	5.99 (0.85)	4.70 (0.47)	5.07 (0.70)	4.36 (0.48)	11.01 (0.26)	11.44 (0.10)	10.79 (0.28)	11.17 (0.16)	11.83 (0.28)	11.61 (0.52	10.80 (0.33)	10.69 (0.19)
N (%)	0.58 (0.05)	0.61 (0.07)	0.49 (0.04)	0.53 (0.07)	0.44 (0.03)	0.35 (0.04)	0.50 (0.03)	0.41 (0.02)	0.17 (0.01)	0.18 (0.01)	0.15 (0.01)	0.14 (0.01)	0.22 (0.01)	0.17 (0.02)	0.16 (0.01)	0.20 (0.01)
Fe (%)	0.41 (0.08)	0.41 (0.09)	0.36 (0.09)	0.44 (0.12)	0.27 (0.07)	0.24 (0.07)	0.30 (0.07)	0.29 (0.08)	1.26 (0.04)	1.27 (0.04)	1.15 (0.08)	1.24 (0.05)	1.35 (0.03)	1.23 (0.05)	1.23 (0.05)	1.25 (0.05)
Si (%)	0.75 (0.03)	0.69 (0.02)	0.75 (0.05)	0.81 (0.05)	0.72 (0.24)	0.60 (0.17)	0.93 (0.31)	0.89 (0.39)	4.61 (0.11)	4.88 (0.25)	4.65 (0.10)	4.57 (0.18)	4.53 (0.09)	4.43 (0.10)	4.47 (0.07)	4.63 (0.08)
Ca (%)	40.24 (2.76)	38.69 (2.60)	41.04 (2.83)	37.46 (1.74)					1.07 (0.31)	0.65 (0.13)	0.68 (0.15)	0.59 (0.09)				
TP (mg/kg)	1080.70 (180.49)	933.56 (159.13)	553.44 (65.13)	583.44 (153.84)	741.56 (144.85)	457.56 (64.89)	698.33 (115.76)	540.56 (86.01)	340.56 (25.78)	414.36 (29.74)	329.22 (34.53)	275.44 (30.82)	960 (232.79)	428.33 (40.26)	381.67 (41.53)	443.11 (40.21)
AP (mg/kg)	12.4 (6.20)	7.12 (3.12)	10.1 (4.79)	5.42 (2.65)	20.13 (3.66)	10.42 (2.58)	19.84 (5.13)	15.56 (5.01)	62.98 (11.15)	81.54 (12.33)	75.52 (21.82)	43.01 (9.56)	158.22 (15.59)	85.87 (16.78)	54.94 (9.80)	67.93 (10.65)
Mn (mg/kg)	140.44 (9.02)	148.89 (10.84)	143.44 (9.84)	144.22 (11.72)	145.33 (11.74)	119.13 (9.48)	161.22 (13.04)	154.78 (16.80)	237.11 (20.54)	276.64 (16.56)	231.11 (24.81)	203.44 (17.94)	199.67 (12.09)	181.78 (11.77)	225.11 (32.37)	231.22 (14.54)
Zn (mg/kg)	19.46 (2.47)	21.70 (3.34)	17.32 (1.90)	19.49 (3.50)	21.18 (2.03)	18.16 (1.64)	24.58 (2.88)	23.19 (3.68)	43.48 (0.93)	47.35 (2.10)	43.36 (2.48)	42.72 (0.88)	54.32 (2.01)	55.32 (6.39)	47.33 (1.67)	48.08 (0.85)
Cu (mg/kg)	12.52 (0.65)	12.52 (0.64)	12.52 (0.65)	12.23 (0.64)	11.80 (0.52)	10.55 (0.56)	11.84 (0.79)	10.88 (0.55)	7.83 (0.32)	8.88 (0.44)	8.19 (0.41)	7.20 (0.58)	9.73 (0.36)	9.32 (0.67)	10.29 (0.34)	10.12 (0.13)
Exchangeable K (cmol(+)/kg)	0.16 (0.05)	0.12 (0.04)	0.17 (0.05)	0.13 (0.04)	0.20 (0.05)	0.15 (0.02)	0.22 (0.06)	0.18 (0.05)	1.68 (0.15)	1.71 (0.20)	1.04 (0.11)	1.33 (0.18)	2.55 (0.28)	2.17 (0.32)	1.17 (0.15)	1.70 (0.20)
Exchangeable Mg (cmol(+)/kg)	0.23 (0.04)	0.18 (0.03)	0.21 (0.03)	0.19 (0.04)	0.44 (0.08)	0.33 (0.05)	0.19 (0.02)	0.17 (0.02)	8.62 (0.73)	7.91 (0.99)	9.40 (0.90)	10.27 (0.72)	7.79 (0.67)	9.87 (0.62)	8.23 (1.04)	6.63 (1.07)
Exchangeable Na (cmol(+)/kg)	0.15 (0.01)	0.13 (0.01)	0.144 (0.01)	0.16 (0.02)	0.15 (0.01)	0.15 (0.01)	0.13 (0.01)	0.13 (0.01)	1.45 (0.19)	2.23 (0.26)	0.86 (0.12)	1.26 (0.15)	1.16 (0.17)	2.24 (0.32)	0.68 (0.12)	1.14 (0.21)
CEC (cmol(+)/kg)	6.94 (1.25)	7.29 (0.99)	7.57 (1.25)	7.58 (1.33)	8.12 (1.53)	6.62 (1.14)	8.28 (1.26)	7.32 (1.31)	29.64 (0.85)	28.75 (1.05)	29.49 (1.04)	30.48 (0.94)	26.40 (0.89)	24.61 (0.98)	25.03 (0.69)	26.88 (0.82)
EC (μS/cm)	128.67 (11.13)	146.11 (10.02)	129.00 (10.70)	136.11 (10.64)	157.44 (26.63)	145.02 (23.83)	137.88 (12.62)	142.94 (21.15)	728.22 (77.26)	985.82 (126.43)	502.89 (62.49)	613.33 (97.83)	596.78 (61.07)	861.00 (116.47)	486.56 (40.65)	533.67 (61.37)

* PCT, PCS, IPT, and IPS microsites refer to palm circle topsoil (0–15 cm), palm circle subsoil (15–30 cm), inter-palm top soil (0–15 cm), and inter-palm subsoil (15–30 cm), respectively. All the numbers presented in the table were means of 9 replicates and the numbers in the bracket were standard error. ^§^ Abbreviations of different chemical parameters: pH (measured in 1 M potassium chloride), OM (organic matters by loss-on-ignition), N (nitrogen), Fe (iron), Si (silica), TP (total phosphorus), AP (available phosphorus), Mn (manganese), Zn (zinc), Cu (copper), K (potassium), Ca (calcium), Mg (magnesium), Na (sodium), CEC (cation exchange capacity), and EC (electrical conductivity).

## Supplementary Materials

The following are available online at https://www.mdpi.com/2079-7737/9/12/424/s1, Figure S1: Soil sampling time points: (**A**) Samples collected on May 2018 from four separate microsites of Blenheim and Bernam plots; (**B**) samples collected on December 2018 from a composite of 4 microsites for Blenheim and Bernam plots. Abbreviations for the four microsites: PCT—Palm circle topsoil; PCS—Palm circle subsoil; IPT—Inter-palm topsoil; and IPS—Inter-palm subsoil, Figure S2: Rarefaction curves of the samples for 16S- (rarefied to sequences) and 18S- (rarefied to sequences) targeted amplicon sequencing: (**A**) Rarefaction curves constructed with observed features and (**C**) rarefaction curves constructed with phylogenetic distance (Faith’s PD tree) for prokaryotic dataset; (**B**) rarefaction curves constructed with observed features and (**D**) rarefaction curves constructed with Faith’s PD tree for the eukaryotic dataset. Error bars indicate the standard error, Figure S3: Alpha-diversities of prokaryotic and eukaryotic communities for soil samples collected from four different microsites for Bernam and Blenheim soil types: (**A**) Observed features; and (**B**) Faith PD, Figure S4: Taxaplots of the top 10 most abundant prokaryotic (**A**,**B**) and eukaryotic (**C**,**D**) taxa for all the sequenced samples from Blenheim (BL) and Bernam (BE) soils at phylum- (**A**,**C**) and genus- (**B**,**D**) levels. Samples labeled with BL or BE abbreviations only refer to the pooled samples from the four microsites: These samples were collected in May 2018. The remaining samples were collected in December 2018. Abbreviations for the four microsites: PCT—Palm circle topsoil; PCS—Palm circle subsoil; IPT—Inter-palm topsoil; and IPS—Inter-palm subsoil, Table S1: Rainfall records for the year 2017, 2018, and 2019 (monthly and total annual rainfalls) for Blenheim Estate, Table S2: Fertilizers input and information for the years 2018 and 2019, Table S3: Soil physical properties and texture of Blenheim and Bernam soil types collected from four different microsites in May and December 2018.
