# Supplementary material for: Determining Soil Microbial Communities and Their Influence on Ganoderma Disease Incidences in Oil Palm (Elaeis guineensis) via High-Throughput Sequencing
Source: Biology (Basel). 2020 Nov 27;9(12):424. doi: 10.3390/biology9120424 (PMC7760618; doi:10.3390/biology9120424)
Supplement: Supplementary file 1 [file biology-09-00424-s001.zip › Supplementary Biology/Table S1.docx]

**Table S1:** Rainfall records for year 2017, 2018 and 2019 (monthly and total annual rainfalls) for Blenheim Estate.

| **Year** | **Monthly Rainfall Records (mm)** | | | | | | | | | | | | |
| --- | --- | --- | --- | --- | --- | --- | --- | --- | --- | --- | --- | --- | --- |
|  | **Jan** | **Feb** | **Mar** | **Apr** | **May** | **Jun** | **Jul** | **Aug** | **Sep** | **Oct** | **Nov** | **Dec** | ***Total*** |
| 2017 | 345 | 137 | 78 | 62 | 311 | 170 | 112 | 203 | 129 | 126 | 463 | 168 | ***2304*** |
| 2018 | 233 | 50 | 103 | 153 | 108 | 151 | 50 | 93 | 165 | 287 | 152 | 254 | ***1799*** |
| 2019 | 53 | 67 | 124 | 152 | 166 | 151 | 18 | 105 | 98 | 290 | 272 | 158 | ***1654*** |
