# Supplementary material for: Determining Soil Microbial Communities and Their Influence on Ganoderma Disease Incidences in Oil Palm (Elaeis guineensis) via High-Throughput Sequencing
Source: Biology (Basel). 2020 Nov 27;9(12):424. doi: 10.3390/biology9120424 (PMC7760618; doi:10.3390/biology9120424)
Supplement: Supplementary file 1 [file biology-09-00424-s001.zip › Supplementary Biology/Table S2.docx]

**Table S2:** Fertilizers input and information for year 2018 and 2019.

| **Fertilizers (kg/ha/year)^§^** | **2018** | **2019** |
| --- | --- | --- |
| Nitrogen (N) | 108 (March & June) * | 94 (March & July) |
| Phosphorus (P) | 51 (May) | - |
| Potassium (K) | 288 (April & August) | 144 (April) |

**^§^**Total fertilizers applied for the respective year, and the fertilizers were either applied once or twice a year (as indicated by the months in the bracket).

*Months in bracket indicate the months respective fertilizers were applied.
