# Supplementary material for: Determining Soil Microbial Communities and Their Influence on Ganoderma Disease Incidences in Oil Palm (Elaeis guineensis) via High-Throughput Sequencing
Source: Biology (Basel). 2020 Nov 27;9(12):424. doi: 10.3390/biology9120424 (PMC7760618; doi:10.3390/biology9120424)
Supplement: Supplementary file 1 [file biology-09-00424-s001.zip › Supplementary Biology/Table S3.docx]

**Table S4.** Soil physical properties and texture of Blenheim and Bernam soil series collected from four different microsites in May and December 2018.

| **Physical parameters** | **Blenheim (*Typic Quartzipsamment*)** | | | | | | | | **Bernam (*Typic Endoaquepts*)** | | | | | | | |
| --- | --- | --- | --- | --- | --- | --- | --- | --- | --- | --- | --- | --- | --- | --- | --- | --- |
|  | **May 2018** | | | | **Dec 2018** | | | | **May 2018** | | | | **Dec 2018** | | | |
|  | **PCT** | **PCS** | **IPT** | **IPS** | **PCT** | **PCS** | **IPT** | **IPS** | **PCT** | **PCS** | **IPT** | **IPS** | **PCT** | **PCS** | **IPT** | **IPS** |
| Clay | 0.67 (0.33) | 0.89 (0.59) | 0.67 (0.47) | 2.22 (1.51) | 0.44 (0.29) | 1.33 (0.94) | 1.11 (0.68) | 0.67 (0.67) | 43.11 (0.82) | 44.00 (1.14) | 43.11 (0.95) | 44.00 (1.15) | 42.00 (1.11) | 44.67 (1.60) | 41.78 (1.47) | 43.78 (0.85) |
| Fine Silt | 6.44 (0.59) | 6.67 (1.63) | 7.11 (1.60) | 6.89 (1.89) | 7.56 (1.14) | 7.56 (0.87) | 9.78 (1.61) | 9.11 (1.67) | 29.78 (1.27) | 28.73 (1.05) | 30.00 (1.60) | 29.78 (0.97) | 34.89 (1.11) | 34.44 (0.99) | 35.11 (0.95) | 34.44 (0.87) |
| Coarse Silt | 2.11 (0.35) | 2.22 (0.46) | 2.11 (0.42) | 2.67 (0.55) | 1.78 (0.43) | 1.22 (0.40) | 1.78 (0.36) | 1.67 (0.24) | 18.33 (1.12) | 16.73 (1.18) | 16.33 (0.58) | 16.33 (0.97) | 17.00 (0.60) | 15.78 (0.40) | 17.56 (0.44) | 16.44 (0.38) |
| Fine Sand | 6.56 (1.18) | 6.33 (0.87) | 6.33 (0.73) | 7.56 (0.88) | 5.00 (0.87) | 5.22 (1.41) | 5.44 (1.17) | 5.11 (0.82) | 7.22 (1.33) | 8.18 (0.98) | 9.11 (1.68) | 7.67 (1.04) | 4.22 (0.57) | 3.89 (0.82) | 4.22 (0.81) | 4.00 (0.99) |
| Coarse Sand | 83.89 (3.39) | 84.00 (3.13) | 83.89 (2.85) | 81.00 (4.55) | 85.22 (2.54) | 84.89 (3.00) | 81.89 (3.43) | 83.44 (2.77) | 1.56 (0.38) | 2.36 (0.72) | 1.44 (0.34) | 2.22 (0.49) | 1.89 (0.26) | 1.22 (0.28) | 1.33 (0.17) | 1.33 (0.47) |
| Texture | Coarse sand to loamy coarse sand | | | | | | | | Silty clay to silty clay loam | | | | | | | |

^*^PCT, PCS, IPT, and IPS microsites refer to as palm circle top soil (0-15 cm), palm circle sub-soil (15-30 cm), inter-palms top soil (0-15 cm), and inter-palms sub-soil (15-30 cm), respectively.
