# Supplementary figures and images for: Determining Soil Microbial Communities and Their Influence on Ganoderma Disease Incidences in Oil Palm (Elaeis guineensis) via High-Throughput Sequencing
Source: Biology (Basel). 2020 Nov 27;9(12):424. doi: 10.3390/biology9120424 (PMC7760618; doi:10.3390/biology9120424)

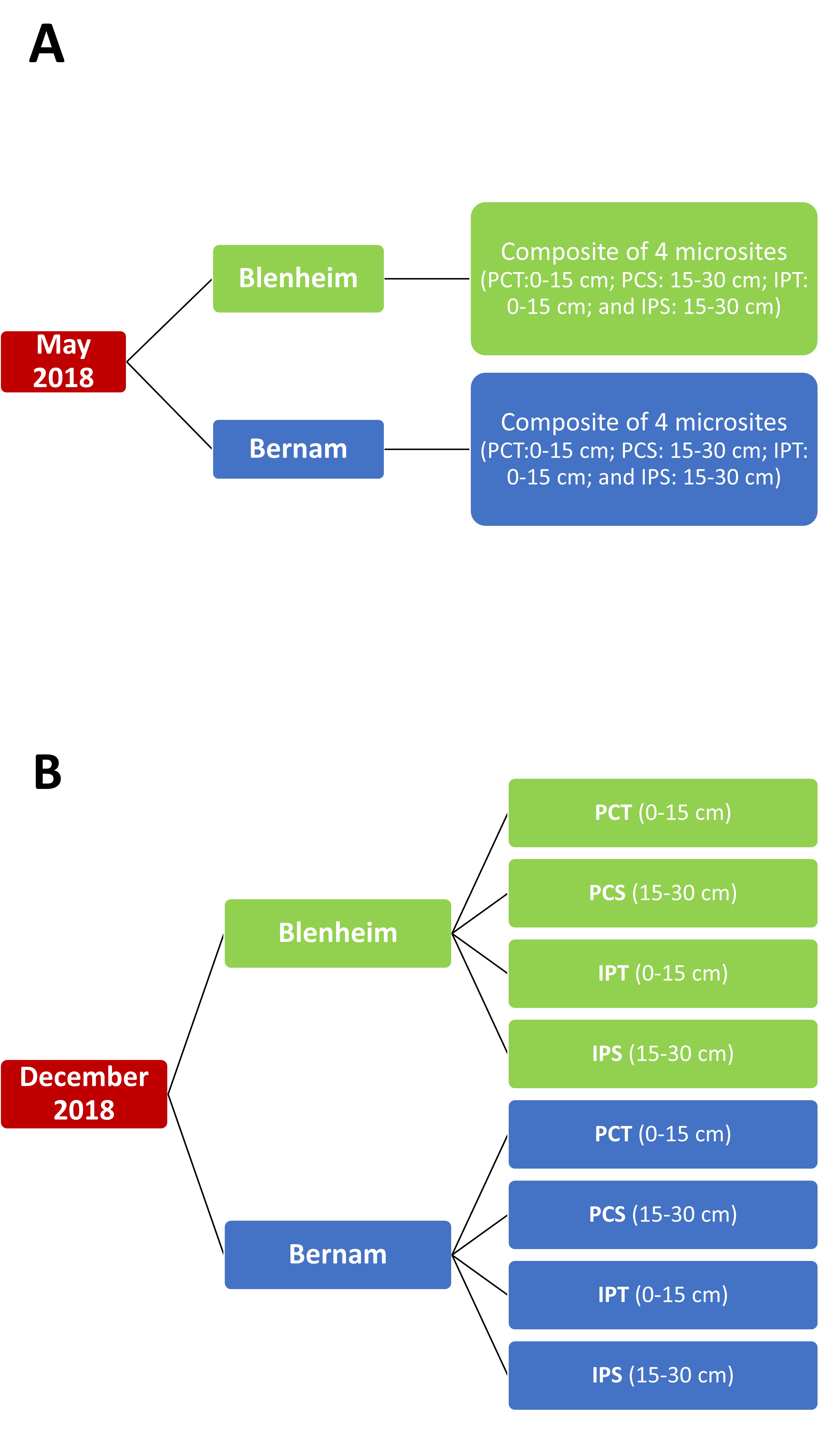

Supplement: Supplementary file 1 [file biology-09-00424-s001.zip › Supplementary Biology/Figure S1.jpg]

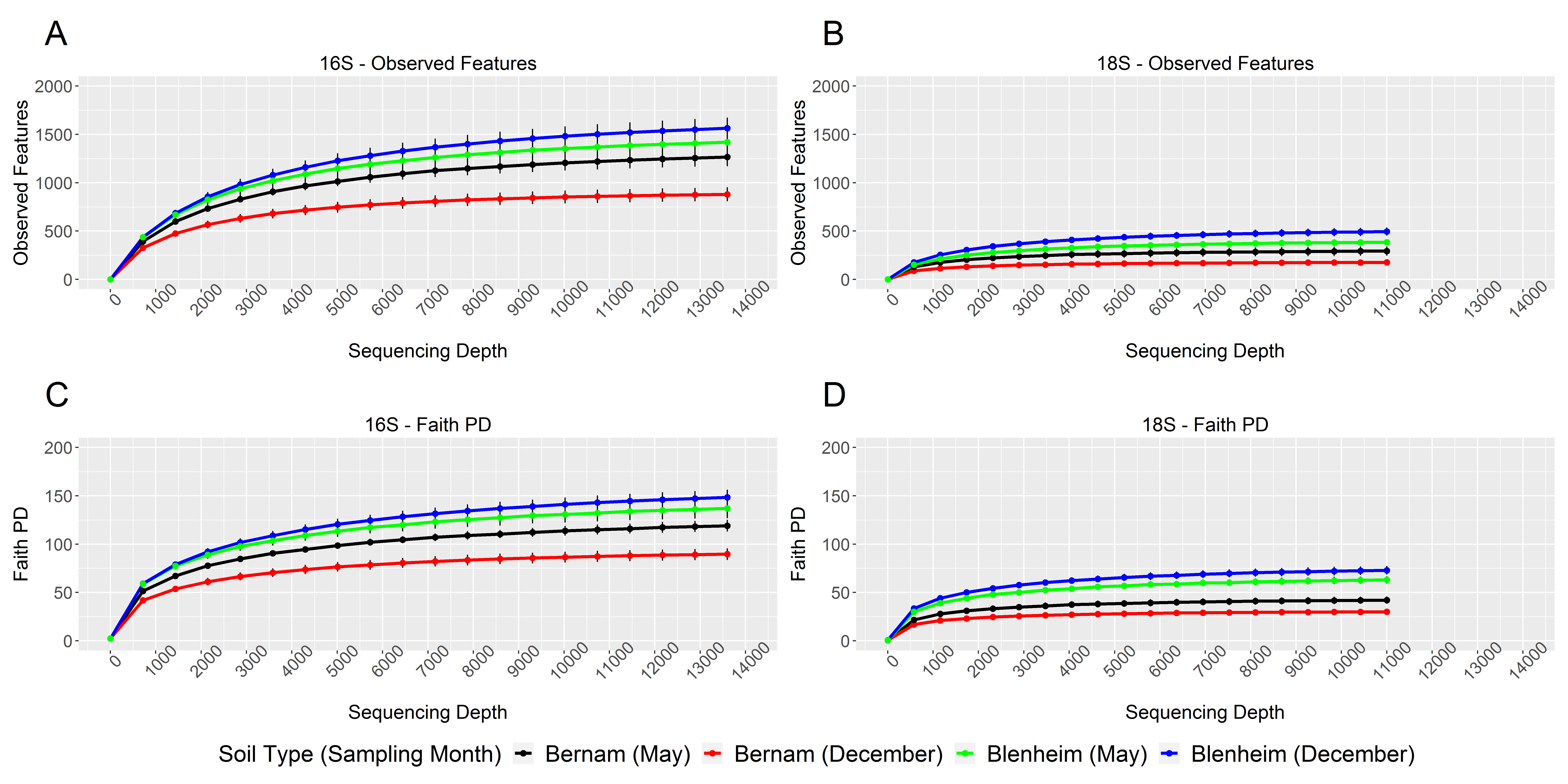

Supplement: Supplementary file 1 [file biology-09-00424-s001.zip › Supplementary Biology/Figure S2.tiff]

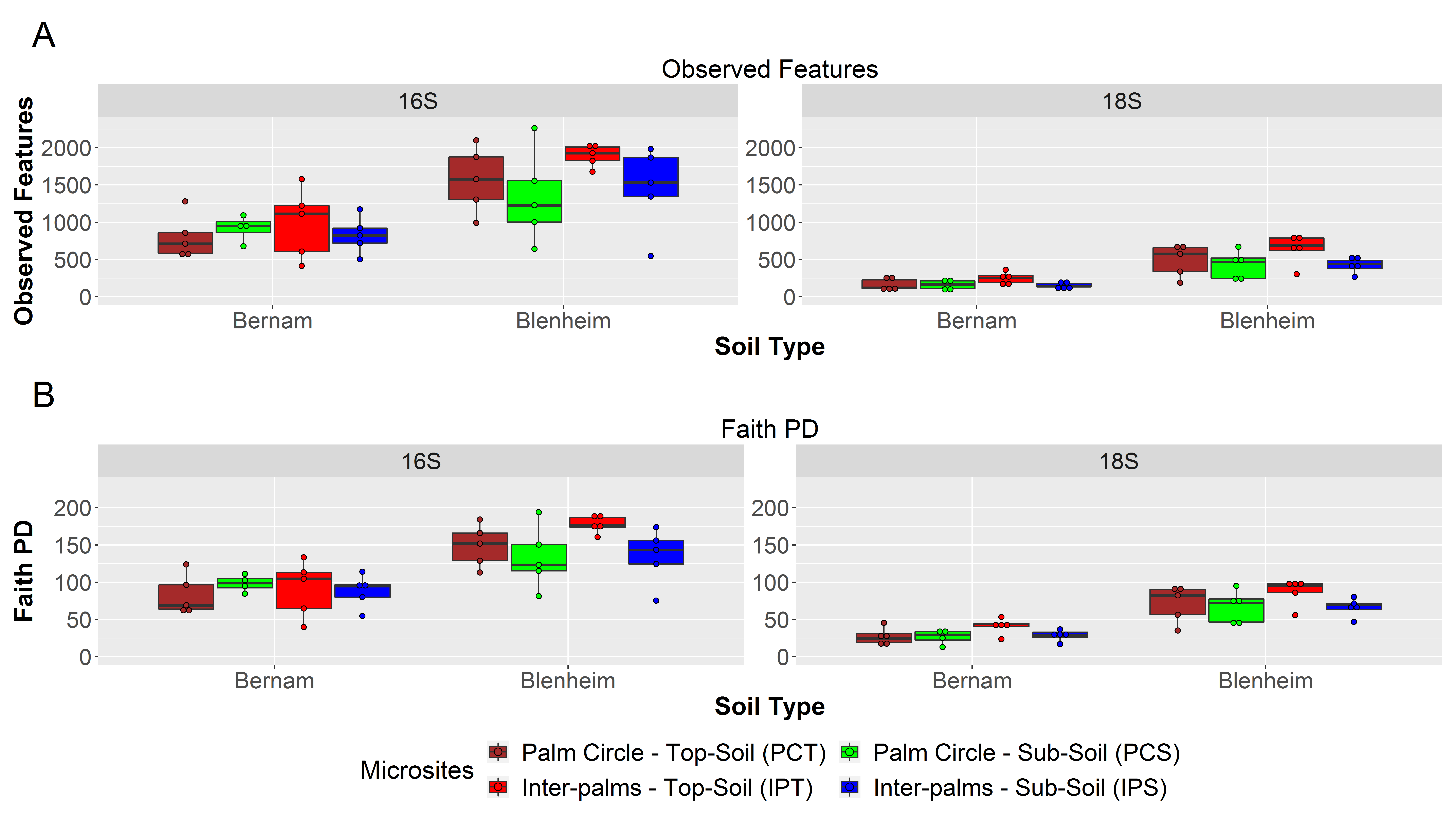

Supplement: Supplementary file 1 [file biology-09-00424-s001.zip › Supplementary Biology/Figure S3.tiff]

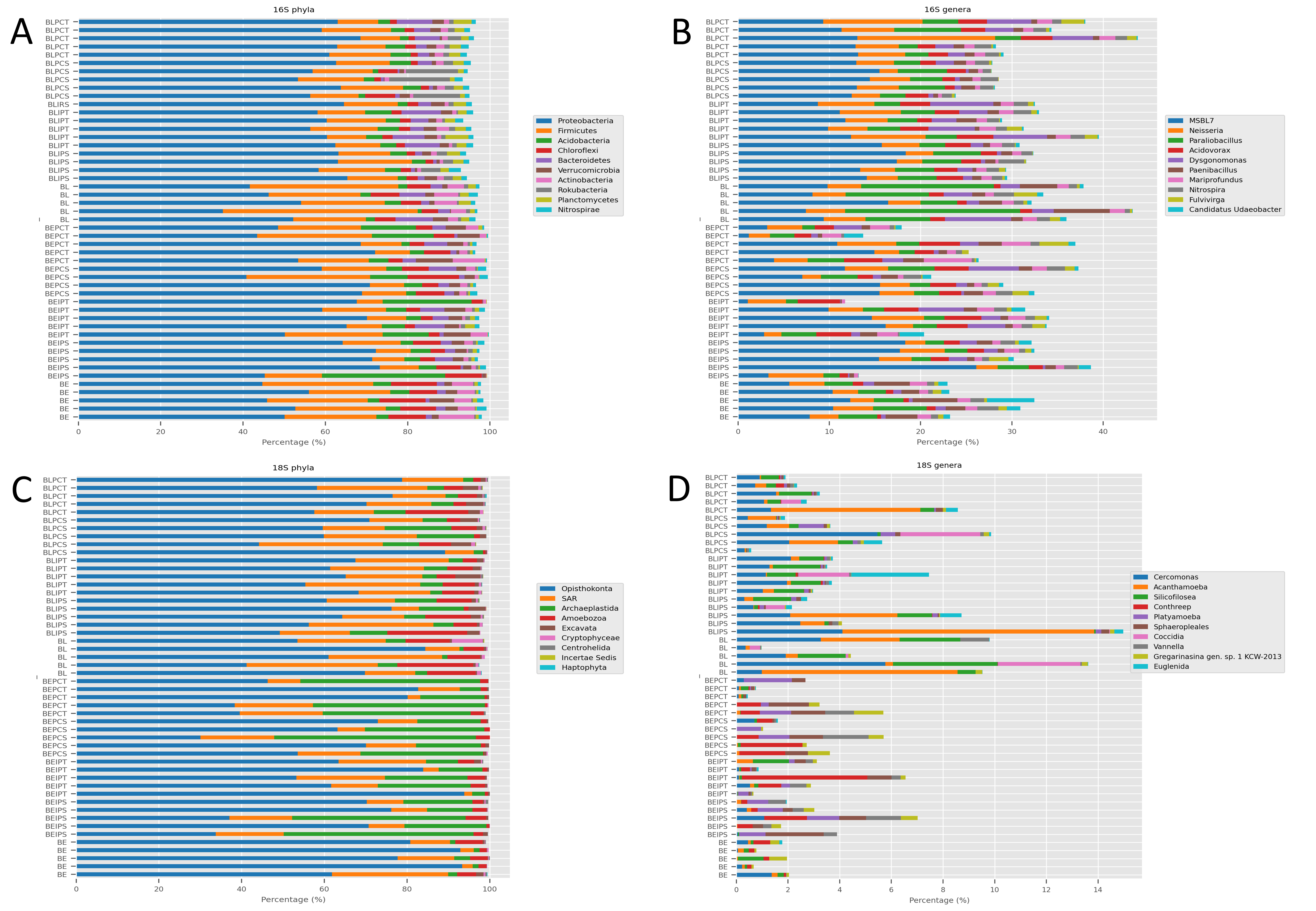

Supplement: Supplementary file 1 [file biology-09-00424-s001.zip › Supplementary Biology/Figure S4.tif]
